# Electrochemical determination of T_2_ toxin by graphite/polyacrylonitrile nanofiber electrode

**DOI:** 10.1002/fsn3.2097

**Published:** 2021-01-08

**Authors:** Mona Moradi, Maryam Azizi‐Lalabadi, Parisa Motamedi, Ehsan Sadeghi

**Affiliations:** ^1^ Department of Chemical Engineering‐ Food Sciences Kermanshah Science and Research Branch Islamic Azad University Kermanshah Iran; ^2^ Research Center for Environmental Determinants of Health (RCEDH) Kermanshah University of Medical Sciences Kermanshah Iran

**Keywords:** biosensor, electroanalysis, modified electrodes, T_2_‐toxin, voltammetric techniques

## Abstract

Fabricating graphite electrode corrected with nanofiber by electrospinning as a considerable procedure for utilization in the fluid materials, milk, and syrup for detection of T_2_ mycotoxin is a significant technique. The modern biosensor was fabricated at normal degrees of room and utilized via buffer Britton–Robinson (B‐R) in pH = 5 to refine the chemico‐mechanical specifications. The electrochemical manner of the modified surface was surveyed using the scanning electron microscopy (SEM), cyclic voltammetry (CV), square wave voltammetry (SQWV), electrochemical impedance spectroscopy (EIS), and differential pulse voltammetry (DPV). The corrected electrode displayed a linear reply to T_2_ toxin in two distinct concentration ranges of 30–100 nM with correlation coefficients of 0.99. The greatest signals in the square wave spectrums for the B‐R buffer created on the uttermost signals of the obtained streams were pH = 5 and 0.5 M of KNO_3_ for T_2_ toxin. The modified electrode has a big signal, broad dynamic concentration and high sensitivity and selectivity.

## Graphical abstract

Mycotoxins produced by fungal and microbial growth from food and agriculture products. T_2_ Mycotoxin is a naturally occurring mold byproduct of *Fusarium* spp. Electrospinning has considerable application to detect several T_2_ toxin.

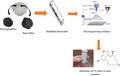

## 1 INTRODUCTION

Mycotoxin is toxic chemical substances which fabricated via certain mold with teratogenic and carcinogenic impact on human health. There are many such compounds, but only a few of them are regularly found in food (Boutrif, 1995; da Rocha et al., 2014; De Ruyck et al., 2015). T_2_ toxin is a *Fusarium* spp mold byproduct that is poisonous to humans and animals. It produces a clinical disorder called alimentary toxic aleukia as well as a crowd of indications connected to tissues as varied as skin, airway, and stomach. T_2_ toxin can be attracted by people skin (Babakhanian et al., 2015; Boonen et al., 2012). Though no considerable impacts are anticipated after cutaneous contact in agricultural or habitable areas, local skin influences cannot be excluded. So, skin contact with T_2_ toxin must be confined. These substances are totally very stable and are not decomposed in preservation of food, baking, producing etc. several Fusarium poisons are related with specified kinds of grain cereal. For instance, T_2_ toxins are often associated with oats, maize, barley, and wheat. These toxins do not decompose at warm condition. This material has an epoxide loop and various acetyl and hydroxyl agents on its side chains. These properties mostly cause biological activity of the product as well as produce more poison. T_2_ toxin has a good ability to prevent nucleic acids fabrication and can cause apoptosis. The poisoning of T_2_ toxin is because of its 12,13‐epoxy loop (Li et al., 2011). Epoxides are generally poison substance which can connect to nucleophiles and lead to more enzymatic responses. The activity of epoxide groups caused to reacted with materials and cellular component such as nucleic acids and proteins (Wan et al., 2015).

Popular method for quantitative recognition of mycotoxin includes the following: high‐performance liquid chromatography coupled with ultraviolet detector (HPLC‐UV), gas chromatography (GC) coupled with electron capture (ECD), flame ionization (FID), enzyme‐linked immunosorbent assays (ELISA), and thin‐layer chromatography (TLC) (Meneely et al., 2011; Pascale, 2009; Rodriguez‐Mozaz et al., 2007). These techniques have some disadvantages including complicate specimen procurement, being expensive, requiring lots of the time and solvent and professional operator (Manasa et al., 2019). Hence, based on the influences of T_2_ toxin versus human safety, there is a powerful requirement to expand a trusty, simple, susceptible, and efficient and valuable analysis procedure to classified mycotoxin in the food substances (Babakhanian et al., 2015; Rodriguez‐Mozaz et al., 2007).

Electrochemical biosensors is a new functional method which was developed as a promising strategy to evaluate the bio‐molecules (Meneely et al., 2011; Rahmani et al., 2009). This biosensor work based on the interaction between bio‐substances and analytes, which are the most common conventional biosensor (Husain et al., 2016). Electrochemical biosensors are considerably noteworthy due to the small size, simpleness function, high sensivity and selectivity, cheapness, biodegradability, transportability, capability to continuous respond, and preparing an exact information (D'Souza et al., 2017). Electrochemical biosensors have a wide application in farming (Karimi‐Maleh et al., 2019), food technologies (Tahernejad‐Javazmi et al., 2019), and medical fields (Khodadadi et al., 2019). Therefore, detection of cost‐effective electrochemical biosensors with the aims of increasing electrocatalytic sensitivity and declining potential oxidation is a novel and promising method. Recently, biosensor with modified surface electrode has been done as a new method for improving the limits of detection of materials (Shetti, Malode, Nayak, Aminabhavi, et al., 2019). Chemically modified electrodes such as carbon paste electrodes have gained the great attention because of producing the high surface, decline the fouling impact and facilitated of surface renewal (Shikandar et al., 2018). Thus recognition of biomolecules can be conducted by different chemically modified sensors. It should be note that surface correction can be applied in the shape of thick or thin layer and or mono films. By this process, modifier practices as an intermediary for quick electron movement among analytes and modified surface. Large modified surface increase sensivity and electrocatalytic activities and the analyte can diffuse more rapid on surface of electrode (Yola, 2019; Yola & Atar, 2019a, 2019b). Shetti et al. (2020) designing the skin‐patchable electrodes for wearable biosensors which can measure heart beat and blood pressure (Kim et al., 2018) as well as permit convenience examination of essential bio‐chemical markers by sweat, saliva, tears etc (Amjadi et al., 2016). Therefore, thus, the noninvasive recognition by using of this process high degree of health can be provided (Shetti, Bukkitgar, Reddy, et al., 2019). Another modified carbon paste electrode for detection of ferulic acid including screen‐printed electrodes (Blanco et al., 2015), screen‐printed carbon electrodes, graphite pencil electrodes (David et al., 2016), laccase‐modified graphite electrode, and electrochemically reduced graphene oxide‐based electrochemical sensor (Liu et al., 2014) etc. To get favorable conclusion, complex process for production of electrode, low pH, and precise evaluating of the electrode stability are important factors. Shetti, Malode, Nayak, Reddy, et al. (2019) designed a new biosensor with silica gel as a modifier in order to increase the electrocatalytic efficiency of paracetamol. The silica gel has three dimensional structure and considered as a strong inorganic materials, as well as has high surface area. The silanol agent (Si – OH) in this compounds exert great ion exchange in them in a bit alkaline condition (Zaazaa et al., 2015). As results, diffusion rate increase and more binding sites are created for analytes (Shetti, Bukkitgar, Reddy, et al., 2019).

Monoclonal antibody label‐free immunoassay in contrast to immune sensor through enzyme label has industrialized to straight identify toxins via substantially altering the immune compound (Tang et al., 2010; Vidal et al., 2011). The substantial benefits for the biologically sensors remains susceptibility, its transportability, and simple equipment. Electrochemical immune sensors have a great application in portable devices in two aspects: direct and sandwich methods (Catanante et al., 2016; Hosseini et al., 2013; Liu et al., 2012). Sandwich methods have high sensitivity and selectivity in detection of T_2_ toxin by hydrodynamic fluid stream (Okuno et al., 1987). Also, electro‐chemiluminescence (ECL) can efficiently detect T_2_ toxin because of high sensitivity, simple instrumentation, controlling ability of ECL, and low cost (Chu et al., 2012; Lin et al., 2006).

**FIGURE 1:**
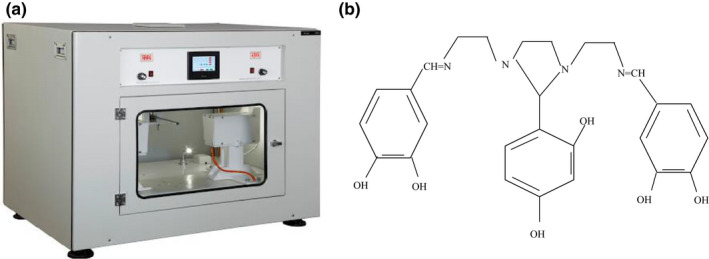
(a) The structural formula for the synthesized component. (b) The image of the electrospinning setup

Electrospinning is a modern procedure with important function in corrected electrodes to discover T_2_ toxin (Chu et al., 2012; Samadian et al., 2017). Electrospinning is a simple, and cost‐effective technique which can made fibers and particles by vast surface, flexibility, better morphologically, and mechanical properties. In the electrospinning procedure, a big voltage is exerted to a polymeric matrix to remove the surface stress. By vaporizing the solvent, the electrospun micrometer‐nanometer fiber is formed (Figure 1a; Mohammadi et al., 2018). Electrospinning has attracted significant attention thanks to its easy production procedure and a variety of appropriate substances (Shaulsky et al., 2017). Different electrospun nanomaterial sensors such as resistant, electrochemical, fluorescent, acoustic wave, colorimetric, and photoelectric sensors have been designed (Cai et al., 2018; Zhang et al., 2017). The electrochemical sensor has several benefits such as high precision, simple and easy equipment; accordingly they have good applications in ultrasensitive discoveries. Polyacrylonitrile (Pan) can fabricate nanofibers by easy electrospun technique. Pan can be straightly applied as an electrode substance (Costentin & Savéant, 2017; Gordon et al., 2017; Nataraj et al., 2012; Wu et al., 2017). Thus, this research assessed a modern, rapid, and simple electrospun method for detection of T_2_ toxin in the food compounds fabricated via electro doping of the incorporated materials (Figure 1b) within the electrospun Pan fibroid composite. Fabricated materials have practical units like hydroxyl group, carboxyl group, oxygen group, and fragrant loops in its network that increase the transition for electron alongside the fibroid composite (Zhang et al., 2017; Zhang et al., 2014). To confirm the electrochemical process of T_2_ toxin in the contaminated food samples, we can use methods like cyclic voltammetry (CV), electrochemical impedance spectroscopy (EIS), and differential pulse voltammetry (DPV), which can approve the morphological properties of T_2_ toxin (Adriano, 2017; Tomac & Šeruga, 2017). Graphene (G) and its derivatives have been used as carbon nano‐materials in electrochemical sensors and fluorescence quencher in optical sensors (Babakhanian et al., 2015; Chen et al., 2012; Molina et al., 2016). The main objective of this research was to detect an efficient solution to define T_2_ toxin in carbon components by correcting the area of the graphite electrode along with nanofibers applying electrospinning.

## 2 EXPERIMENTAL

### 2.1 Reagents and chemicals

T_2_ toxin as tested sample, KI, KNO_3_, NaNO_3_, NaCl, and KCl salt as a sponsor electrode, CH₃COOH, H_3_PO_4_, H_3_BO_3_, and NaOH as the primary material for buffer preparation are required. All chemical substances were bought from Merck and Sigma‐Aldrich corporations (Parzhak Shimi laboratory). Nanofibers were applied to modify the electrode area. Pan, C_5_H_8_O_2_, and H_2_SO_4_ as an auxiliary factor were applied by nanofibers.

### 2.2 Equipments

Electrochemistry tests were done by an Auto lab PGSTAT101 and NOVA.1.11 software. A conservative 3 electrode method was used to integrate an occupied modified electrode as an electrospun G/Pan‐synthesized constituent electrode, an orientation electrode as a soaked Ag/AgCl electrode, and a counter electrode as a graphite electrode. The pH capacities were presented by a 781 Metrohom pH meter prepared with a joint glass electrode. The surface morphology was calculated by an SEM (Philips XL30, Philips Panalytical, California). In this method, firstly, the samples were covered with gold and then SEM was acted at 5 kV.

### 2.3 Procedures

#### 2.3.1 Characterization of nanofiber‐corrected electrode

**FIGURE 2:**
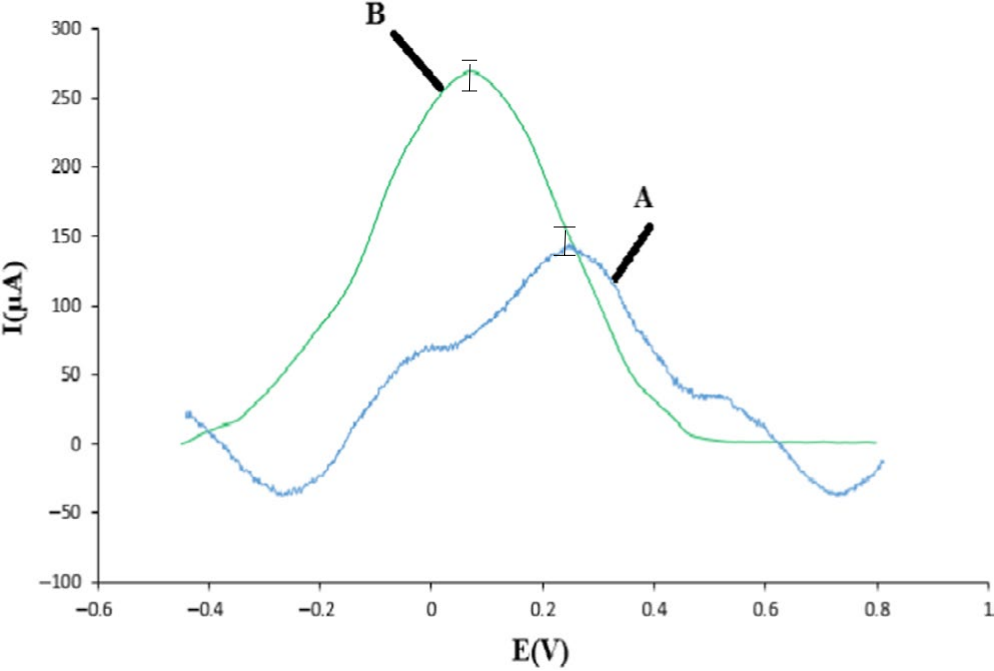
Cyclic voltammogram during different stages of address layer, (a) bare graphite electrode, (b) graphite/nanofiber modified electrode at the presence of B‐R buffer with pH = 5 and 30 μM T_2_ toxin. (Scan speed conditions = 100 mV/s.)

To provide the corrected electrode, the electrospinning method was applied. At first, the exterior of un‐corrected graphite electrode was varnished fine up to it was without of chemical contamination. Next, electrospinning procedure with the volume of 0.0008 ml/min was used to produce the nanofibers containing surface modifier (Bukkitgar et al., 2015; Guha et al., 2014). The best situations in electrospinning device were used the 25 kV voltage, spinning period for 20 min, page gap of 25 cm and moisture amount of 56% (Figure 2).

To prepare T_2_ toxin samples, distilled deionized water was applied for all samples overall the trials. B‐R buffer matrix was provided via incorporation 0.2 M NaOH _(aq)_ to the matrix comprising 0.04 M H_3_BO_3 (aq)_, CH₃COOH _(aq)_, and H_3_PO_4 (aq)_ to create pH = 3–12. Finally, the matrix were polluted by a various amounts of T_2_ toxin (0.5–300 nM).

To procurement nanofibers as electrode corrector cover; electrospinning procedure were performed as previously mentioned for the corrected electrode. Pan along with nano‐clay substance was applied to produce a layer of nanofibers, for this manner syringe pump operated as the fiber sprayer. The best situations for nanofibers film on a graphite electrode area were the 25 kV voltage, spinning period for 20 min, page gap of 25 cm and moisture amount of 56%. In addition, C_5_H_8_O_2_ (7%) and H_2_SO_4_ (5%) were used as a fiber couplers. At last, the fabricated nanofibers were exposed to couplers steam for 20 min, eventually the substances were washed with sulfate buffer 5 times for 7 min each time (Pascale, 2009).

#### 2.3.2 Preparation of T_2_ toxin sample

A modified electrode was applied to measure T_2_ toxin in the contaminated samples. Afterward confirming the samples, they were analyzed for T_2_ toxin detection. Firstly, 9.5 ml B&R buffer thru pH = 5, 0.5 μm KNO_3_ salt, 10 μl based on contaminated samples by various amounts of T_2_ toxin at nano‐molar scales (0.5–300 nm) were occupied in attendance of the corrected electrode and square wave spectrums. Findings were applied as a calibration diagram to measure the amount of T_2_ toxin in the contaminated samples (Catanante et al., 2016; Li et al., 2011).

#### 2.3.3 Voltammetry procedure

The public recommendations applied to catch the square wave voltammetry allied to the outside of graphite/nanofiber‐corrected electrode were as follows:

Firstly, 10 milliliter of B‐R buffer at pH = 5 also a proper content of standard T_2_ toxin matrix—that can create a linear concentration from 0.5 to 100 μl—were incorporated to the 25 ml cell for the Auto lab machine and conveyed to the electrochemical vial of the Auto lab. With using a square wave voltammogram, the potentials were obtained since (−0.4) v to (+0.8) v. Entire analysis was done in the normal temperature (Babakhanian et al., 2015).

#### 2.3.4 pH analysis

The manner which is applied for assessing the influence of pH continuously the respond for nanofiber‐corrected graphite electrode in each analyze were as bellows:

Ten milliliter of B‐R buffer (pH = 3–12) and 30 μM of T_2_ toxin were incorporated to the 25‐ml tube. The matrix were agitated greatly and conveyed to the electrochemical vial of the Auto lab device. Next, the matrix with various pH amounts was occupied from a cyclic voltammogram. It should be noted that the best pH in these analyses were the pH which fabricated the highest flow velocity (Adriano, 2017).

#### 2.3.5 Salt concentration investigation

In these procedures, the impact of electrolyte salt amount on the sensor reply was evaluated applying 30 mM of T_2_ toxin at pH 5. The important factor in this manner was the highest signal which caught up by the sensor (Adriano, 2017).

## 3 RESULTS AND DISCUSSION

### 3.1 Evaluations of nanofiber‐corrected electrode

The nanofiber‐corrected electrode was applied to determine T_2_ toxin in the contaminated products. The corrected electrode was gotten at the existence of 30 μl of T_2_ toxin and silver chloride (standard electrode) in the CV. This procedure enhance the peak altitude relevant to the oxidization of T_2_ toxin on the corrected electrode area in the matrix eventually, by using of this manner T_2_ toxin can be detect along with designed sensor (Figure 2). This outcome is in accordance with the findings of moradi et al. (Moradi et al., 2020; Palmisano et al., 1981).

### 3.2 Influence of scanning electron microscopy from corrected graphite electrode area

**FIGURE 3:**
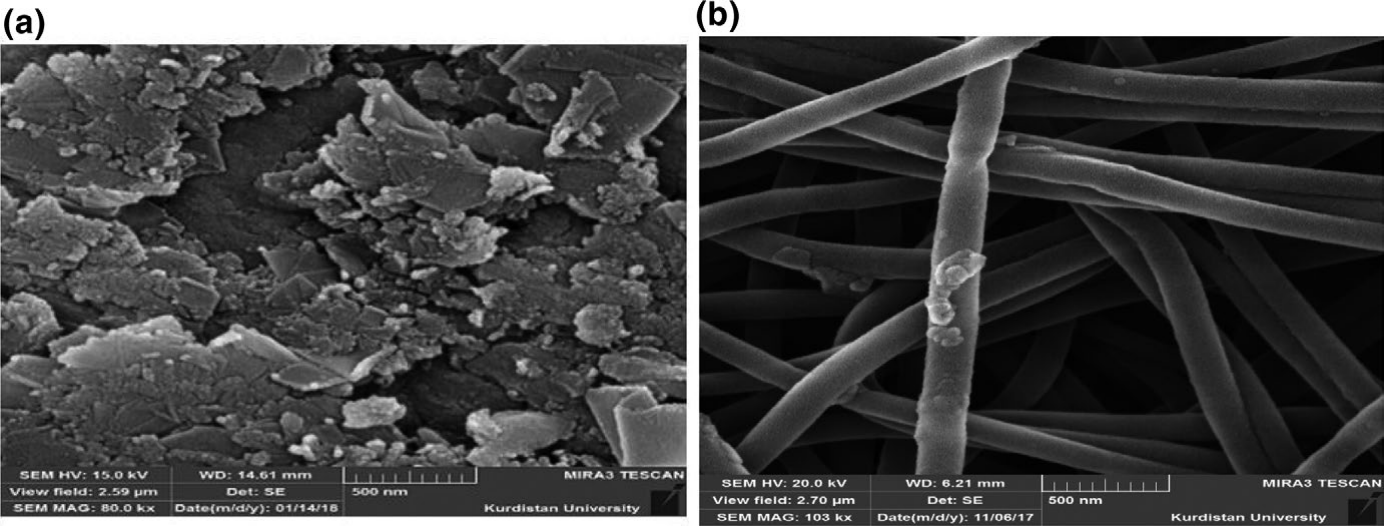
*SEM* image of modified graphite electrode with nanofiber in 133 nm (a) bare graphite (b) modified graphite with nanofiber

The SEM patterns were caught from the area of un‐corrected graphite and nanofiber‐corrected graphite (Figure 3). For un‐corrected graphite electrode, an unsuitable flat surface was seen (Figure 3a). By correcting the graphite electrode area, a superficial thru nano‐scale parts of smaller than 100 nm into diameter were formed, that covered the external adjusting units in theirs constructions (Figure 3b) and could result in a rise in nanometric superficial accessible for oxidation and reduction responses. Furthermore, in the nanofiber‐corrected electrode area, the threads with 133 nm diameter were seen, that could be applied as the dynamic electrocatalytic area used for different tests.

### 3.3 Impact of scan speed

**FIGURE 4:**
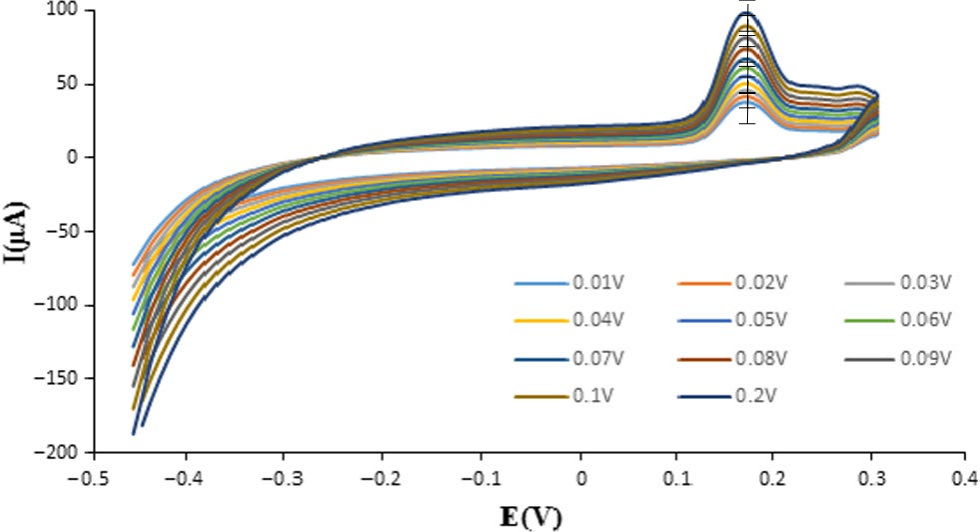
Investigating the effect of SEM by cyclic voltammetric in the presence of modified graphite electrode by B‐R buffer with pH = 5 and 30 μM of T_2_ toxin

The impact for SEM was evaluated in the existence of the corrected electrode at 10–100 mV/s and optimum chemical situations. Then, 10 milliliter of B‐R buffer in pH = 5 and 50 μM for T_2_ toxin in the existence of corrected graphite electrode and optimum chemical substances were carried into the vial. The compounds were surveyed in the existence of the proper sensor in the potential extend. According to the results, 100 mV/s was selected as the suitable speediness for any sample (Figure 4; Bukkitgar et al., 2016; D’Souza et al., 2015).

### 3.4 Impact of pH

**FIGURE 5:**
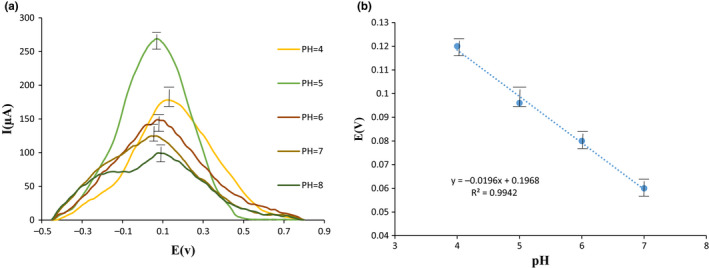
(a) Square wave voltammogram of 30 μM T_2_ toxin on the surface of the G/PAN nanofiber modified electrode at pH 3–12, Britton–Robinson (B‐R) buffer solution (b) linear graph of pH change of 30 μM T_2_ toxin on the surface of the G/PAN nanofiber modified electrode

The influence of pH matrix thru the sample on the cathodic peak of the square wave spectrum at the existence of 30 μM T_2_ toxin for the corrected electrode was assessed at pH = 3–12 in B‐R buffer (Figure 5a and b). High‐level acid/alkaline situation, that is associated to pH = 3–12, intrusion of H‐ (pH = 3) and OH‐ ions (pH = 12) and its assault into the electrode area lead the altering of analyte peak and subsequently eliminated the peak since the pH schedule. Figure 5 displays that T_2_ toxin cathode peak is associate with the pH variations proton receiver agents on the T_2_ toxin structure. The optimized signals pro the square wave spectra in the B‐R buffer according to the highest signal of the obtained stream were pH = 5 for T_2_ toxin. As well as, 10 milliliter of B‐R buffer were determined such as a suitable buffer content. An amount more than 10 ml did not influence the increasing manner of the obtained signal. The pH diagram at pH = 4 into 7 showed a linear manner with the (−19) mV slope for the pH, which proposed the T_2_ toxin reaction on the area of the corrected electrode were a proto/electron manners (Figure 5a and b) (Moradi et al., 2020). The pH chart slopes could be applied to define the electron convey rate by the below formulation:

ΔE/pH=0.0591/n→0.0196=0.0591/n→≅3

pH amounts also powerfully influenced the oxidizing possibility of T_2_ toxin, and enhancing of pH, can decrease oxidation rate to negative amounts (D'Souza et al., 2015; Shetti et al., 2009). As a result, a great linear connections was established among the pH and oxidizing rate.

### 3.5 Influence of salt amount

Choosing appropriate electrolyte in voltammetric responses is extremely significant. The applications of electrolytes such as enhancing the matrix conductivity, regulating the ionic intensity, declining the remaining flow, enhancing the oxidation through faraday flows, decreasing the analyte, and raising the selectivity.

**FIGURE 6:**
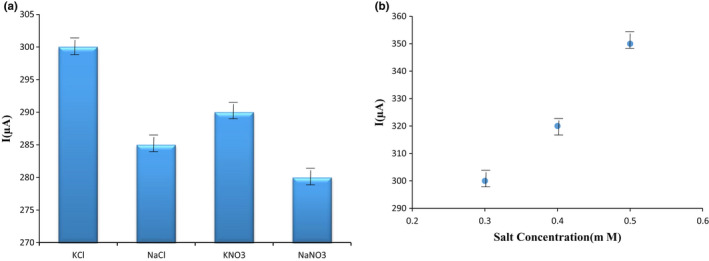
(a) The impact of different salt concentrations with 30 μM T_2_ toxin on the surface of the G/PAN nanofiber modified electrode to determine the effective salt concentration in electron transfer, (b) the effect of different concentrations of KNO_3_ with 30 μM T_2_ toxin on the surface of the G/PAN nanofiber modified electrode to recognize the effective salt concentration in electron transfer as a supporter electrolyte

Thus, impact of electrolyte salts was evaluated in the mentioned trials. Various amounts of backer electrolytes including KCl, KNO_3_, NaCl, NaNO_3_, and KI were assessed. The volume among 0.3 and 0.5 M was regarded as the backer electrolyte volume. The findings displayed which 0.5 M of KNO_3_ as a backer electrolyte was propered to define T_2_ toxin at the existence of the optimal electrode. The reason for this choice was the most signal obtained, as seen in Figure 6a and b.

### 3.6 Calibration diagram in the matrix

**FIGURE 7:**
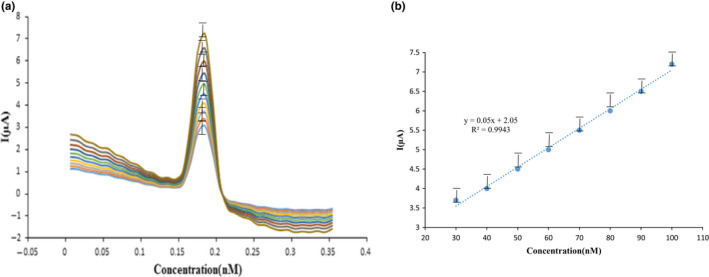
(a) Differential pulse voltammetry (DPV) techniques of the electrochemical behavior of T_2_ toxin over the concentration range 30–100 nM at the presence of B‐R buffer with pH = 5, (b) linear chart of the effect of different concentrations on the transition flow with compressibility coefficient = 0.99

In the appropriate chemico‐mechanical situations, various matrix of T_2_ toxin were collected at the variety of 0.5–550 nM. By using advanced seeking in the variety of 0.0–0.4 V, the voltammetric spectrum of the square wave was obtained and the calibration chart of T_2_ toxin was drawn at the existence of the corrected electrode. The flow‐concentration diagram was taken in linear areas, from 30 to 100 nM (Figure 7a and b). Consequently, the correlation coefficient for whole areas was described to be 0.99. As observed in Figure 5, the content of the conveyed flow was increased via raising the analyte volume, which demonstrated enhancing in the electrode area at the existence of T_2_ toxin in the bed fluid and also show a flow enhance from the exterior of the corrected electrode within the procedure.

Via applying the information gotten from the standard chart and its chart formulation, the limit of quantitation (LOQ) and limit of detection (LOD) were determined. The outcomes of flow/potential and flow/concentration curves were observed in Figure 5. Also, the LOQ and LOD amounts were 30 and 10 nM, respectively. Soleimany et al. (2012), used the liquid chromatography tandem mass spectrometry process to detect aflatoxins B_1_ and B_2_, ochratoxin A, zearalenone, deoxynivalenol, fumonisins B_1_ and B_2_, and T_2_ toxins in cereals products. They reported the LOD and LOQ of mycotoxins in the range of 0.01–25 and 0.02–40 ng/g, respectively. Hossain et al. (2018) by surface plasmon resonance biosensor assay detected the amount of T_2_ toxin in wheat. After preparation of samples and evaluation the amount of T_2_ toxin, the LOD report 1.2 ng/ml. This method was a fast process and has high sensivity for detection of T_2_ toxin in wheat.

$$\text{LOD}=X_{b1}+3S_{b1}$$

$$\text{LOQ}=X_{b1}+10S_{b1}$$

To calculate the LOD, LOQ (three repeated test), the flow cycles were occupied from every concentration and by trebling the quantity of *SD*, and interrupt of calibration curve was analyzed as a middling parameter.

### 3.7 Interference

Assessing the impact of kinds of coexisting and analyte is the most considerable controlled factors. To survey the influence of interfering, the impacts of various volumes of the matrix of possible organic and inorganic substances in simulant on the reply of the corrected electrode with 30 μM T_2_ toxin and modified sensor were investigated (Table 1). In addition, 5% fault in the signals was presented as a border of disorders. Under optimized situations, the findings demonstrated that these substances did not have any specific disorder for the corrected electrode. As a result, it can approve that the corrected electrode has a good capability to recognize T_2_ toxin in the polluting materials.

**TABLE 1:** The effect of interfering organic and inorganic species on the response of the modified electrode to 30 mM T_2_ toxin (three repetitions)

Interfering ion	Tolerated ratio [Interference]/[T2 toxin]	Interfering ion	Tolerated ratio [Interference]/[T2 toxin]
Cu^2+^	270	Mn^2+^	740
Ni^2+^	320	Zn^2+^	580
Fe^2+^	540	Co^2+^	460
K^+^	12,100	l‐Cysteine	1,350
Vitamin B_12_	750	Glucose	1,100
CTAB of thiourea or urea	≥1,250	Vitamin C	290

Abbreviation: CTAB, cetyltrimethylammonium bromide.

## 4 CONCLUSION

In this study, square wave voltammetry procedures were applied as a susceptible electrochemistry manner to discover and evaluate T_2_ toxin. Novel electrodes were fabricated via correcting the electrode area as a layer of nanofibers. These outcomes were distinctly obvious over assessment of the electrode reply in the pollutant compounds. In the corrected graphite electrode, the conductivity and electrochemical features of the electrode area and also the sensitivity of electrode to T_2_ toxin enhanced significantly, mainly in the cyclic voltammetric method. Primary materials such as nanofibers were used to provide the corrected electrode by electrospinning technique. This technique has a great sensitivity and a high capability in evaluating a little content of sample. In square wave voltammetry, due to the possibility of the electrode area, no one of the compounds which can conduct reaction in the compounds affected with the sample discovery. Further, corrected electrodes have different characteristics including big signal‐to‐noise proportion, broad linear reply, great sensitivity, big accuracy and precision, excellent persistence and consistency, observed coating of electrode area, cheapness, and being secure and efficient. Finally, the electrospun sensor fixed into Pan/nanofibers has the supreme applications in discovery of T_2_ toxin in the different compounds.

## CONFLICT OF INTEREST

All authors declare that there is no conflict of interest.

## ETHICAL APPROVAL

This article does not contain any studies with human participants or animals performed by any of the authors.

## Supporting information

Supplementary Material
