# Supplementary material for: Electrochemical determination of T2 toxin by graphite/polyacrylonitrile nanofiber electrode
Source: Food Sci Nutr. 2021 Jan 8;9(2):1171–9. doi: 10.1002/fsn3.2097 (PMC7866594; doi:10.1002/fsn3.2097)
Supplement: Supplementary file 1 — Supplementary Material [file FSN3-9-1171-s001.docx]

Supplementary information

**2.3.6. Device parameters**

Apparatus factors are one of the most significant parameters which must be assessed within the refining of electrode surface. As well as, 30 μM of T_2_ toxin matrix and best chemical situations were used. The reason for the optimum selection of instruments factors were the highest signal flow via the square wave voltammetry procedure. The best amounts were as bellows:

Sweep rate: 0. 6 V

Amplitude: 0.04 V

Frequency: 60 Hz

Voltage step: 0.010 V

**2.3.7. Analysis of statistics**

The statistical analysis of the findings of entire the analyses was conducted via SPSS program (Ver 21.0, IBM; Armonk, NY, USA), applying one-way analysis of variance (ANOVA). The considerable outcomes was done by Duncan's multiple range post hoc tests (P < 0.05) to compare the mean values of responses and all analyses were done in triplicate.
